# Contribution of the Multiplicity Fluctuation in the Temperature Dependence of Phonon Spectra of Rare-Earth Cobaltites

**DOI:** 10.3390/molecules25184316

**Published:** 2020-09-20

**Authors:** Yuri S. Orlov, Alexey E. Sokolov, Vyacheslav A. Dudnikov, Karina V. Shulga, Mikhail N. Volochaev, Sergey M. Zharkov, Nikolay P. Shestakov, Maxim A. Vysotin, Sergei G. Ovchinnikov

**Affiliations:** 1 Kirensky Institute of Physics, Federal Research Center KSC SB RAS, 660036 Krasnoyarsk, Russia; alexeys@iph.krasn.ru (A.E.S.); slad63@yandex.ru (V.A.D.); volochaev91@mail.ru (M.N.V.); zharkov@iph.krasn.ru (S.M.Z.); nico@iph.krasn.ru (N.P.S.); mav@iph.krasn.ru (M.A.V.); sgo@iph.krasn.ru (S.G.O.); 2 Institute of Engineering Physics and Radio Electronics, Siberian Federal University, 660041 Krasnoyarsk, Russia; tabakaewa-karina@mail.ru

**Keywords:** rare-earth cobalt oxides, multiplicity fluctuations, phonon spectra

## Abstract

We have studied, both experimentally and theoretically, the unusual temperature dependence of the phonon spectra in NdCoO_3_, SmCoO_3_ and GdCoO_3_, where the Co^3+^ ion is in the low-spin (LS) ground state, and at the finite temperature, the high-spin (HS) term has a nonzero concentration $n_{HS}$ due to multiplicity fluctuations. We measured the absorption spectra in polycrystalline and nanostructured samples in the temperature range 3–550 K and found a quite strong breathing mode softening that cannot be explained by standard lattice anharmonicity. We showed that the anharmonicity in the electron–phonon interaction is responsible for this red shift proportional to the $n_{HS}$ concentration.

## 1. Introduction

The close-to-spin crossover (SCO) of the high-spin (HS) and the low-spin (LS) terms of a magnetic cation, the energy of the multiplicity fluctuation $\Delta_{S}=E_{HS}-E_{LS}$, is small. The phenomenon of SCO in 3d-metal oxides is usually realized under high pressure [1], while in metal–ligand complexes in an organic matrix, the SCO may be induced by changing temperature [2,3,4,5]. There is also one more group of 3d-metal oxides, the rare-earth cobaltites RCoO_3_ with a perovskite structure, very close to SCO [6,7,8,9,10]. The Co^3+^ ion is in the LS ground state for all rare-earth ions, with very small energy $\Delta_{S}$~100 K for La. More-heavy rare-earth ions with smaller ionic radii have large energy $\Delta_{S}$ [8,11]. With heating, the thermal excitation over the gap $\Delta_{S}$ HS states results in a smooth change in structural, electronic, magnetic and thermodynamic properties; see [12] and the review [13]. Recently, we studied the effect of multiplicity fluctuation in cobalt ions on the crystal structure and magnetic and electrical properties of NdCoO_3_ and SmCoO_3_ [14], in comparison with GdCoO_3_ [12]. The effect of multiplicity fluctuation on lattice vibration has not received much attention in the literature, while the large (about 10%) ionic-radius difference for the HS and LS states certainly may result in a change in the metal–ligand vibrations. Here, we study this effect by optical spectrum measurements in NdCoO_3_, SmCoO_3_ and GdCoO_3_ at different temperatures. We compare optical spectra in two types of samples, polycrystalline ceramics and nanostructured polycrystalline samples.

Usually, the temperature dependence of the phonon frequency in crystals results from the electron–phonon or phonon–phonon interactions (anharmonicity). Volume-expansion measurements in GdCoO_3_ have revealed the noticeable contribution of the anharmonicity above 1000 K (see below). In this paper, we discuss the measured temperature dependence for *T* < 500 K, which may be related to the electron–phonon interaction. We show that the local CoO_6_ breathing mode is strongly renormalized by the HS thermally excited states, and the main effect results from the anharmonicity in the local electron–phonon interaction, which is important due to the large ionic-radius difference for HS and LS states.

The structure of the paper is the following: information on the sample preparation and experimental methods is given in Ch.2; experimental data for the structure and optical spectra at different temperatures are given in Ch.3. The data analysis and theoretical interpretation of the temperature-dependent phonon frequency are presented in Ch.4. Ch.5 contains the conclusions.

## 2. Results

**Figure 1:**
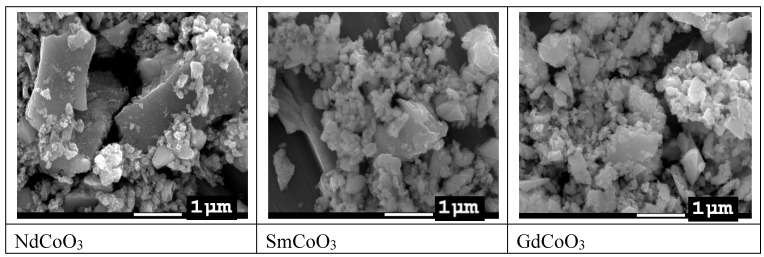
SEM images of nanostructured samples after ultrasonic treatment.

**Figure 2:**
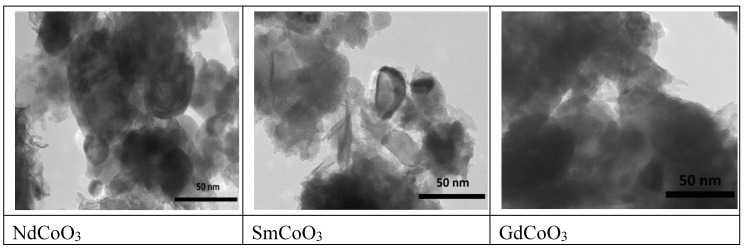
Photos from a transmission electron microscope for nanostructured samples after ultrasonic treatment; scale bar, 50 nm.

The SEM images showed a very broad nanoparticle size distribution (Figure 1). Therefore, for transmission electron microscopy, we used nanoparticles remaining in suspension after exposure to ultrasound; the smallest nanoparticle size, about 10 nm, was obtained for the sample GdCoO_3_ (see Figure 2).

**Figure 3:**
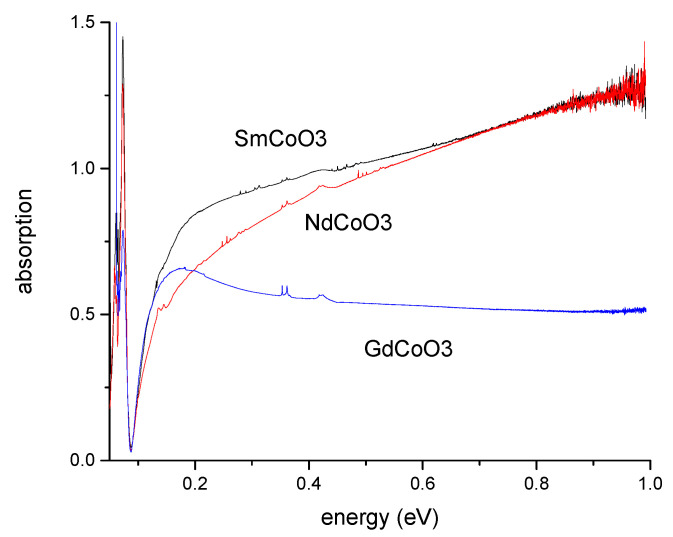
The absorption spectra of NdCoO_3_, SmCoO_3_ and GdCoO_3_, measured at *T* = 300 K.

The optical absorption spectra of NdCoO_3_, SmCoO_3_ and GdCoO_3_ at room temperature are shown in the Figure 3 up to the energy 1 eV. The absorption above 0.1 eV is provided by electrons, and in the following, we will restrict ourselves to phonon absorption in the region of energies less than 0.1 eV.

**Figure 4:**
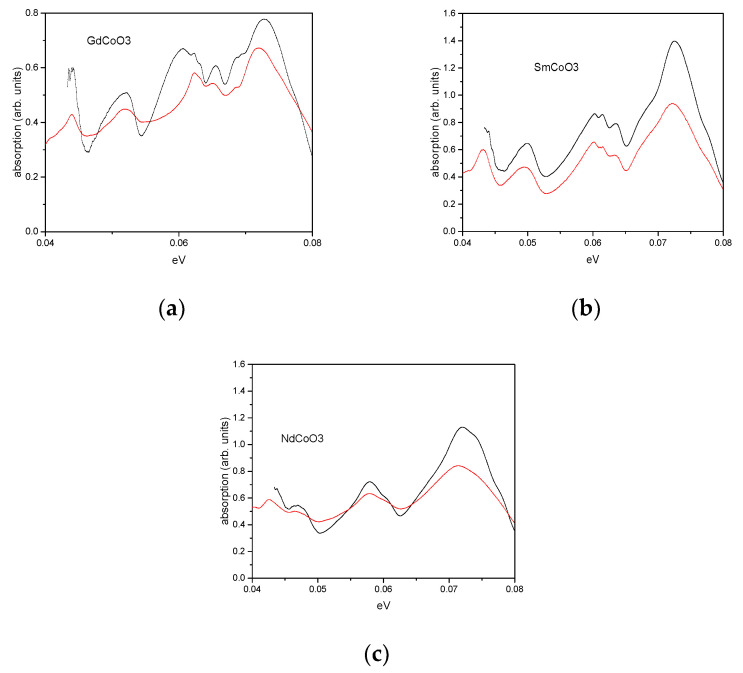
Comparison of absorption spectra of nanostructured (red) and polycrystalline (black) samples at room temperature. (**a**) GdCoO_3_; (**b**) SmCoO_3_; (**c**) NdCoO_3_.

**Figure 5:**
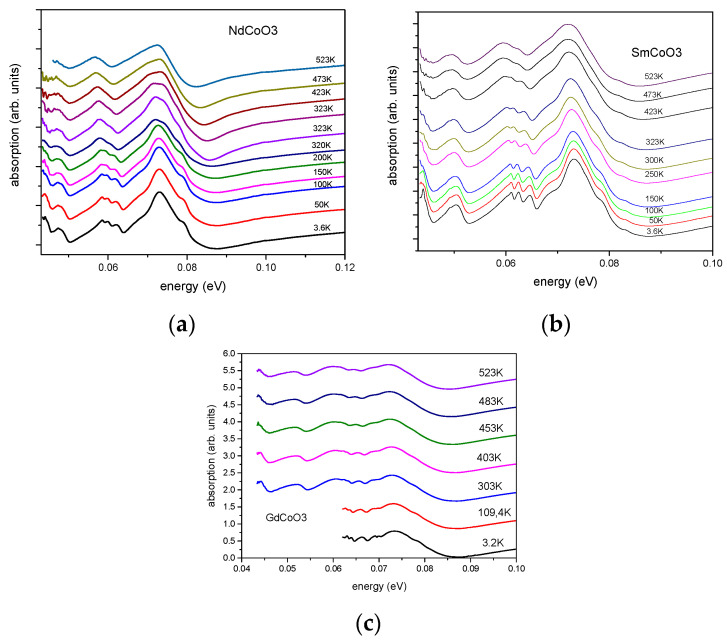
Temperature-dependent infrared absorption spectra for NdCoO_3_ (**a**), SmCoO_3_ (**b**) and GdCoO_3_ (**c**).

In this work, we pay special attention to the higher-energy part of the phonon spectrum (0.04–0.08 eV) (see Figure 4 and Figure 5), in which the breathing vibronic mode is located. The high-energy part of the phonon spectrum reveals the well-resolved vibrational excitations, similar to those observed in [15].

The comparison of the infrared spectra for the nanostructured and polycrystalline samples in Figure 4 indicated the same energies for the vibronic excitations, close to 0.0725 eV at room temperature. The temperature-dependent phonon-absorption spectra are given in Figure 5, which shows that heating from liquid-helium temperature to 523 K shifts the narrow-peak position by about 73 meV in the direction of decreasing energy.

**Figure 6:**
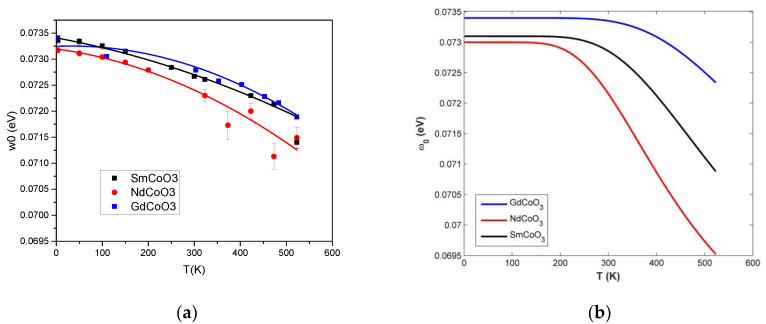
The shift of the breathing-mode peak position for different samples depending on temperature measured (**a**) and calculated in Ch.4 (**b**). The calculations were performed for the following values of the spin gap $\Delta_{S}$: $\Delta_{{\mathrm{NdCoO}}_{3}}=1400$ K; $\Delta_{{\mathrm{SmCoO}}_{3}}=1800$ K; $\Delta_{{\mathrm{GdCoO}}_{3}}=2300\;$ K.

The highest phonon energy peaks that we associated with the breathing mode (see Ch.4 for a discussion) shift their energy for all three compounds (Figure 6). The shift for Nd is maximal, and that for Gd is minimal.

## 3. Discussion

### 3.1. Ab Initio Calculation of Optical Phonons in GdCoO_3_

The optical phonon frequencies were calculated in the framework of the density functional theory (DFT, [16]) implemented in the VASP 5.4.1 program package [17,18]. The exchange-correlation functional of the Perdew–Burke–Ernzerhof formulation [19] was used in combination with the plane-wave basis and the projector-augmented wave (PAW) method [20]. The basis set was limited by the cutoff energy of 520 eV; the integrations over the first Brillouin zone were performed using 8 × 8 × 6 k-point meshes of the Monkhorst–Pack scheme [21].

The crystal structure of GdCoO_3_ in the orthorhombic *Pbnm* phase was fully relaxed to cell size and atom positions; the resulting lattice parameters were *a* = 5.2284 Å, *b* = 5.4970 Å and *c* = 7.4805 Å which agree with previously reported experimental data [12]. The ground state corresponds to low-spin cobalt cations and 7 μ_B_ magnetic moments on gadolinium, which are antiferromagnetically ordered (E(FM)–E(AFM) = 1.92 meV/atom). The interatomic force constants were calculated by the finite displacement method within a unit cell and several larger supercells so that the resulting phonon frequencies were converged within 0.05 THz for the calculation cell size. The optical phonon frequencies (*q* = 0) were found using the PHONOPY code [22]. While the CoO_6_octahedra in the *Pbnm* phase are distorted and tilted away from the high-symmetry case, the pure stretching and bending octahedral modes are mixed. To find the breathing mode, all vibration eigenvectors were projected on the direction of uniform elongation of the Co-O bonds. The mode with the highest contribution to the breathing motion (80%) corresponds to the mode of the highest frequency of 17.01 THz. This B_2g_ mode corresponds to breathing oscillations of neighboring octahedra in the counter-phase. It is worth noting that the amplitude of the apical oxygen is 84% higher than the amplitudes of the basal atoms, which is connected with the difference in Co-O bond lengths: 1.936 Å and 1.968 Å, respectively.

The vibration modes with frequencies close to the breathing one are B_3g_, counter-phase octahedral stretching (16.54 THz), which is Raman-active as well as a breathing mode [23], and B_2u_, in-phase mixed octahedral bending/stretching (16.28 THz), which is IR-active. The next 19 modes in the range of 13.8–15.9 THz are of stretching and bending natures, with some contribution of Co-atom displacements. The rotational motions of the octahedra appear below a small frequency gap with the B_3g_mode at 11.81 THz. A comparison of the phonon energies measured at *T* = 3.2 K and those calculated can be seen in Table 1.

### 3.2. Effect of Electron–Phonon Interactionon Temperature Dependence of the Phonon Energy

To describe the effect of multiplicity fluctuations on the phonon-absorption spectrum, we write the Hamiltonian of the electron and phonon subsystems and their interaction in the form:

(1) $$\hat{H}=\underset{i}{\sum }\left(\frac{1}{2}k{\hat{q}}_{i}^{2}+\frac{{\hat{p}}_{i}^{2}}{2M}\right)-\frac{1}{2}V_{q}\underset{\left\langle i,j\right\rangle }{\sum }{\hat{q}}_{i}{\hat{q}}_{j}-\underset{i}{\sum }\left(g_{1}{\hat{q}}_{i}+g_{2}{\hat{q}}_{i}^{2}\right)\left({\hat{n}}_{i,HS}-{\hat{n}}_{i,LS}\right)$$

Here, the first term contains the energy of local symmetric vibrations of the cation–anion complex (which we consider as a unit cell and, hereinafter, we will call the SCO complex), while the second and third describe the elastic interaction of cations on neighboring lattice sites and electron–vibron interactions, respectively. The parameters $g_{1}$ and $g_{2}$ are denoted for linear and quadratic electron–vibron interactions within the SCO complex (MeO_6_ octahedra), k is the elastic coupling constant, M is the anion mass, $\hat{q}$ is the normal coordinate operator of the Me-O breathing vibration, $\hat{p}$ is the corresponding momentum operator, and $V_{q}$ is the parameter of interatomic elastic coupling. The Me-O bond length is equal to $l=l_{0}+\langle \hat{q}\rangle$, where $l_{0}$ is the equilibrium bond length. Since the ionic radii of the cations in the LS and HS states differ quite strongly (the difference is about 10%), it is necessary to take into account not only linear but also quadratic terms in the electron–vibron interaction. For example, in many Fe oxides (Fe_2_O_3_, FeBO_3_ etc.), the spin crossover under high pressure at critical value *P*_c_~50 GPa is the first-order phase transition accompanied by the volume change ~10% [1]. In conventional condensed-matter theory the linear on *q* electron–phonon interaction is the harmonic contribution while the quadratic one describes the anharmonicity effects. For example, it contains an emission of two phonons simultaneously in the interaction process [24].

We want to comment on the electron–vibron interaction part; typically, it is given by the product of an oscillator normal coordinate $\hat{q}$ and electronic density operator ${\hat{n}}_{e}=\underset{p}{\sum }c_{p}^{+}c_{p}$, where $c_{p}\left(c_{p}^{+}\right)$ are electron-annihilation (creation) operators [25,26]. In our two-level model of the two ionic terms, the HS and the LS, at each cation, the electron-density operator is written via multielectron occupation number operators ${\hat{n}}_{i,HS}$ and ${\hat{n}}_{i,LS}$. The mean value $\left\langle {\hat{n}}_{i,HS}\right\rangle$ gives the probability of the HS term, while $\left\langle {\hat{n}}_{i,LS}\right\rangle$ provides the probability of the LS state; together, $\langle {\hat{n}}_{i,HS}\rangle +\langle {\hat{n}}_{i,LS}\rangle =1$. For the precise definition of these operators, we use the multielectron projection Hubbard *X* operators [27,28]. Nevertheless, in this paper, we use the simplest approximation decoupling the anharmonic interaction term ${\hat{q}}_{i}^{2}\left({\hat{n}}_{i,HS}-{\hat{n}}_{i,LS}\right)\to {\hat{q}}_{i}^{2}\left(\left\langle {\hat{n}}_{i,HS}\rangle -\langle {\hat{n}}_{i,LS}\right\rangle \right)$, so only the HS occupation numbers $n_{HS}=\langle {\hat{n}}_{i,HS}\rangle$ and similar $n_{LS}=\langle {\hat{n}}_{i,LS}\rangle$ enter the theory. Contrarily, the dynamics under optical pumping require treating the complicated algebra of X operators [28]. As in the conventional two-level model, these occupation numbers are determined by their energies $E_{HS}$ and $E_{LS}$ and degeneracy factors $m_{HS}$ and $m_{LS}$:

$$n_{HS}=\frac{m_{HS}e^{-E_{HS}/k_{B}T}}{m_{LS}e^{-E_{LS}/k_{B}T}+m_{HS}e^{-E_{HS}/k_{B}T}}$$

$$n_{LS}=1-n_{HS}.$$

For Co^3+^, the LS state is nondegenerate, $m_{LS}=1$, and the HS state with spin 2 and pseudo-orbital momentum 1 has $m_{HS}=15$. Finally, the difference in the signs of the HS and LS contributions in the electron–vibron interaction reflects the difference in the ionic radii; a more compact LS ion will result in a bond-length contraction, while a larger HS- ion will increase the l value.

The Hamiltonian (1) in our approximation can be transformed into the form

(2) $$\hat{H}\approx \underset{i}{\sum }\left(\frac{1}{2}\left[k+2g_{2}\left(1-2n_{HS}\right)\right]{\hat{q}}_{i}^{2}+\frac{{\hat{p}}_{i}^{2}}{2M}\right)-\frac{1}{2}V_{q}\underset{\left\langle i,j\right\rangle }{\sum }{\hat{q}}_{i}{\hat{q}}_{j}+g_{1}\left(1-2n_{HS}\right)\underset{i}{\sum }{\hat{q}}_{i}$$

**Figure 7:**
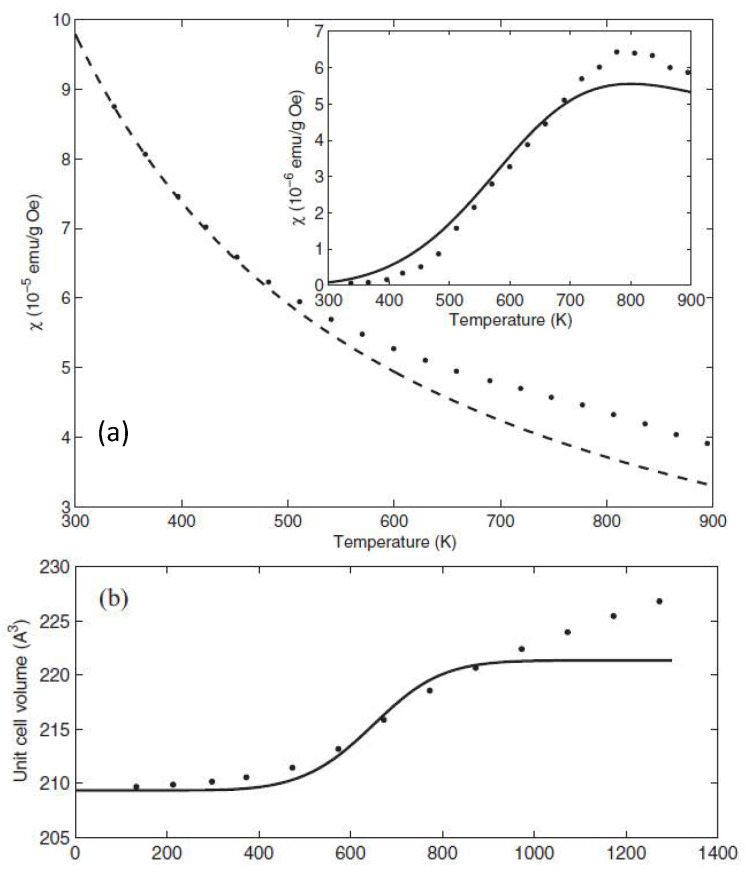
The temperature dependence of (**a**) the magnetic susceptibility; dots show the measured values; the dashed line is for the Gd^3+^ Curie–Weiss contribution (with the Co^3+^high-spin (HS) contribution shown by a solid line in the inset, where the difference of the measured and Gd contribution is shown by dots). (**b**) The volume expansion in GdCoO_3_ from our paper [12]. The solid line in (**b**) corresponds to the calculated low-spin (LS) volume at low *T* and HS volume calculated in harmonic approximation at high *T*; the deviation of the experimental dotted line demonstrates the lattice anharmonicity effect at *T* > 1000K.

From Equation (2), it can be seen that the elastic coupling constants in the LS- ($n_{HS}=0$) and HS- ($n_{HS}=1$) states are equal to $k_{LS}=k+2g_{2}$ and $k_{HS}=k-2g_{2}$, respectively; therefore, the frequencies of the local vibrations differ in the HS- and LS- states: $\omega_{HS}=\sqrt{k_{HS}/M}=\sqrt{\left(k-2g_{2}\right)/M}$; $\omega_{LS}=\sqrt{k_{LS}/M}=\sqrt{\left(k+2g_{2}\right)/M}$. For the selected parameter values from the paper [29] (k=7.5 eV/Å^2^ and $g_{2}=0.75$ eV/Å^2^), we found $\omega_{HS}=0.045$ eV and $\omega_{LS}=0.055$ eV. These are the phonon frequencies in the HS and the LS states for a given set of parameters. For the first-order phase transition under high pressure such as for FeBO_3_, one would find the phonon frequency $\omega_{HS}$ for pressure *P* < *P*_c_ and the frequency $\omega_{LS}$ for pressure *P* > *P*_c_. The typical shift in the Raman spectra of the order 100 sm^−1^ was measured at the spin crossover in Fe_2_O_3_ in the paper [30]. Nevertheless, in LnCoO_3_, there is no sharp spin crossover transition with heating at ambient pressure; the LS state is the ground state with a temperature-dependent admixture of the HS state, so the phonon frequency (given below by Equation (5)) is an average with strong red shift. In Figure 7, we demonstrate the smooth evolution of the magnetic properties and volume expansion of GdCoO_3_ with heating up to 1300 K.

The Hamiltonian (2) may be written in the form $\hat{H}={\hat{H}}_{0}+{\hat{H}}_{1}$, where

(3) $${\hat{H}}_{0}=\underset{i}{\sum }\left(\frac{1}{2}\left[k_{LS}-4g_{2}n_{HS}\right]{\hat{q}}_{i}^{2}+\frac{{\hat{p}}_{i}^{2}}{2M}\right)-\frac{1}{2}V_{q}\underset{\left\langle i,j\right\rangle }{\sum }{\hat{q}}_{i}{\hat{q}}_{j}$$

(4) $${\hat{H}}_{1}=g_{1}\left(1-2n_{HS}\right)\underset{i}{\sum }{\hat{q}}_{i}$$

With the help of standard canonical transformation [31], we can reduce (3) to the form ${\hat{H}}_{0}=\underset{k}{\sum }\omega_{k}\left(b_{k}^{+}b_{k}+\frac{1}{2}\right)$, where for a simple cubic lattice, $\omega_{k}=\sqrt{{\tilde{\omega }}_{0}^{2}\left(1-\frac{V_{q}}{3\tilde{k}}\left(\cos k_{x}+\cos k_{y}+\cos k_{z}\right)\right)}$. Here, $\tilde{k}=k+2g_{2}\left(1-2n_{HS}\right)$,

(5) $${\tilde{\omega }}_{0}=\sqrt{\omega_{LS}^{2}-n_{HS}\frac{4g_{2}}{M}}=\sqrt{\omega_{LS}^{2}-n_{HS}\Delta \omega^{2}}\approx \omega_{LS}-n_{HS}\Delta \omega^{2}/2\omega_{LS}$$

where $\Delta \omega^{2}=\left(\omega_{LS}^{2}-\omega_{HS}^{2}\right)\approx 0.001$ eV. For our samples, the LS phonon frequency at T=0 is about 70 meV, according to Table 1. We should mention that a similar effect of the anharmonicity of the electron–phonon interaction on the optical phonon frequencies has been discussed for the problem of the ferroelectricity in perovskites [32,33], with some hint of high-temperature superconductivity in cuprates [24,34].

Due to the lanthanide contraction, the spin gap value $\Delta_{S}=E_{LS}-E_{HS}$ (the energy interval between the LS and HS states) increases with increasing element number for the rare-earth ions [11]. Using Equation (5), we can estimate the effect of multiplicity fluctuations on the phonon-absorption spectrum with increasing temperature. The right part of Figure 6 shows the calculated temperature dependence of the position of the maximum of the IR absorption spectrum corresponding to the excitation of the breathing mode of the crystal-lattice vibrations for all three compounds. It is seen that the GdCoO_3_ sample has the highest value for the spin gap and ${\tilde{\omega }}_{0}$ over the entire temperature range, as a denser medium, due to the smallest ionic radius of the gadolinium ion. As the temperature rises, the absorption maximum shifts to lower frequencies due to increasing high-spin-state occupation (multiplicity fluctuations), which qualitatively corresponds to the obtained experimental data (Figure 6a).

## 4. Samples and Experimental Methods

The samples LnCoO_3_ were obtained with standard ceramic technology using a stoichiometric amount of high-purity oxides Co_3_O_4_, 99.7% (metals basis, Sigma-Aldrich, St. Louis, MO, USA) and Ln_2_O_3_ (Ln = Nd, Sm, Gd), 99.99% (Rare Metals Plant, Novosibirsk, Russia). To obtain samples with a grain size of 20 to 50 μm, the initial compositions were thoroughly mixed in an agate mortar using ethanol, annealed in air at a temperature of *T* = 1373 K in a corundum crucible for 24 h with a triple grinding–calcining cycle and cooled together with the furnace to room temperature at a speed of 2 K/min. After annealing, the mixture was ground again; tablets were pressed into bars of 5 × 13 × 2 mm^3^, which were then annealed in air at a temperature of 1473 K for 8 h, cooled and ground for measurements.

Additionally, nanoscale samples were prepared to investigate the effect of particle size on optical properties. Powders with a grain size of less than 100 nm were obtained by wet grinding in deionized water using a Pulverisettte 7 premium-line planetary micro mill (Fritsch GmbH, Idar-Oberstein, Germany) in two stages. In the first stage, a powder with a grain size of 20 to 50 μm was loaded into a zirconium dioxide (96.2% ZrO_2_) milling bowl with ZrO_2_ balls 3 mm in diameter and milled for 150 min at 800 rpm. Furthermore, additional grinding was carried out for 30 min at 1100 rpm. After evaporation, the resulting powder was placed in an ultrasonic bath and crushed in ethyl alcohol for 5 min.

Powder X-ray diffraction (PXRD) data for the polycrystalline samples have been discussed recently in [14]. Here, we also present the electron microscopy data. The elemental analysis was carried out with a scanning electron microscope (SEM) with an X-ray spectroscopy detector (EDX, Hitachi TM 3000). The study of the sample’s structural properties was carried out using transmission electron microscopy (TEM) using a Hitachi HT7700 microscope at an accelerating voltage of 100 kV.

The morphology of the samples was investigated with SEM with a JEOL JSM-7001F electron microscope operating at 15 kV. For these measurements, the nanoparticle powder was placed in ethanol and subjected to ultrasonic treatment for 5 min.

In this paper, we measured the NdCoO_3_, SmCoO_3_ and GdCoO_3_ absorption spectra in the infrared (IR) region at temperatures from 3.2 to 523 K. The IR spectroscopy measurements were carried out with the vacuum Fourier Transformation IR spectrometer Vertex 80 v equipped with an RT-DLaTGS detector. Cryogenic measurements were carried out with a cryostat-type OptistatAC-V12 and Temperature Controller ITC503s from Oxford Instruments in the range 3.2–296K. For the temperature region 297–523 K, we used a Variable Temperature Cell 147/QV High Stability Temperature Controller 4000 Series TM fromSpecac Ltd. The sample was prepared by mixing 0.200 g of KBr powder and 0.001 g of crystal mass, to be pressed into a tablet of diameter 13 mm.

## 5. Conclusions

We have demonstrated, experimentally, an unusually strong softening of the optical phonon mode, and we have theoretically related it to fluctuations of the multiplicity that result in a thermally induced partial occupation of the high-spin terms. In the row NdCoO_3_, SmCoO_3_ and GdCoO_3_, the spin-gap value increases [11], where the increase in the HS concentration is the largest for NdCoO_3_ and the smallest for GdCoO_3_. This large phonon-energy shift results from the anharmonic contribution in the electron–phonon interaction. While all the effects of anharmonicity in the lattice are usually related with very high temperature, here, we have revealed that the fluctuations of multiplicity in the electronic system due to anharmonic electron–phonon interaction results in a quite noticeable effect at the moderate temperatures of 300–500K due to small spin-gap values.

Contrary to other materials with SCO with a sharp switching between HS and LS states under high pressure or heating [2,3,4], the rare-earth cobaltites demonstrate a smooth increase in the HS population due to the multiplicity fluctuations.

## Figures and Tables

**Table 1:** Calculated and measured highest-energy optical phonons for GdCoO_3_.

Mode	B_2u_	B_3g_	B_2g_
Energy, theor., meV	67.3	68.4	70.4
Energy, exper., meV	66.3	68.8	73.3
